# Recoverability of *Microcystis aeruginosa* and *Pseudanabaena foetida* Exposed to a Year-Long Dark Treatment

**DOI:** 10.3390/microorganisms11112760

**Published:** 2023-11-13

**Authors:** Hongyu Yan, Mudalige Don Hiranya Jayasanka Senavirathna

**Affiliations:** Graduate School of Science and Engineering, Saitama University, 255 Shimo-Okubo, Sakura-ku, Saitama 338-8570, Japan

**Keywords:** bioreserve, cyanobacterial growth, extensive darkness, photosynthetic ability, stress recovery

## Abstract

Cyanobacteria are a significant primary producer and pioneer species that play a vital role in ecological reconstruction, especially in aquatic environments. Cyanobacteria have excellent recovery capacity from significant stress exposure and are thus suggested as bioreserves, even for space colonization programs. Few studies have been conducted on the recovery capacity after experiencing stress. Long-duration darkness or insufficient light is stressful for photosynthetic species, including cyanobacteria, and can cause chlorosis. Cyanobacterial recovery after extensive exposure to darkness has not yet been studied. In this experiment, *Microcystis aeruginosa* and *Pseudanabaena foetida* were subjected to a year-long darkness treatment, and the change in recovery capacity was measured in monthly samples. Cyanobacterial growth, chlorophyll-a concentration, oxidative stress, and photosynthetic capacity were evaluated. It was found that the rapid recovery capacity of the two species remained even after one year of darkness treatment. However, the H_2_O_2_ content of recovered samples of both *M. aeruginosa* and *P. foetida* experienced significant changes at six–seven months, although the photosynthetic capacity of both cyanobacteria species was maintained within the healthy range. The chlorophyll-a and carotenoid content of the recovered samples also changed with increasing darkness. The results showed that long-term dark treatment had time-dependent effects but different effects on *M. aeruginosa* and *P. foetida*. However, both cyanobacteria species can recover rapidly after one year of dark treatment.

## 1. Introduction

Cyanobacteria are often assumed to be the first oxygenic photosynthetic organisms [1] and have persisted since the Archean Eon [2]. As pivotal primary producers and pioneer species, they exhibit outstanding stress resilience [3]. Scientists have conducted numerous simulations on the response of cyanobacteria to extreme conditions, including polar regions [4], space radiation [5,6], a high-chlorine salt environment in the surface of Mars [7], Martian-like pure CO_2_ atmosphere conditions [8], high temperature [5], and high light [9], in anticipation that it can become a pioneer species to transform outer planets and reserve bioenergy during disasters. Studies have demonstrated that cyanobacteria, especially *Microcystis aeruginosa,* survive in extreme environments, although oxidative stress is produced [10,11].

In addition to their response to extreme conditions, studies on the response of cyanobacteria during storage and transport is essential to determine their potential as pioneer species or reserve bioenergy [12]. A widely known approach to cyanobacterial storage is controlling the temperature and light intensity; cyanobacteria are generally stored in chilled conditions (either on ice or refrigerated) and kept in the dark [13]. It is generally understood that fluctuations in temperature and light intensity can affect metabolic activities in cyanobacteria. For instance, *Pseudanabaena* species exhibit varying chlorophyll-a and 2-MIB (2-Methylisoborneol) production at different temperatures, peaking at 35 °C and diminishing at 15 °C [14]. Cyanobacteria such as *M. aeruginosa* experience oxidative stress under high light conditions [15]. Furthermore, in our previous study [16], *M. aeruginosa* was exposed to extreme light for eight days, followed by an eight-day recovery under optimal conditions. Changes in response to light exposure and postexposure recovery ability were observed. The results showed that *M. aeruginosa* could recover rapidly from high light intensity stress within the period [16]. Conversely, some studies indicate that a short period of darkness, up to 30 days, harms the growth of cyanobacteria; dramatic changes occur in the growth rate, organic secretion, redox state, and pH of cyanobacterial cells [17,18,19].

Numerous studies have explored the variation of cyanobacteria in different storage conditions, such as low temperatures and light exclusion [20,21,22]. However, less attention has been paid to cyanobacterial recovery after prolonged periods of darkness, especially due to storage, transportation, or even during space transport for colonization [20]. A recent study has shown that proper cold storage induces vernalization and accelerates the recovery of *M. aeruginosa*. Samples stored at 4 °C and 10 °C exhibited a significantly enhanced growth rate compared to those at 28 °C [21]. Some researchers have suggested that the recovery of cyanobacteria is affected by combined conditions of long-term low temperature and dark storage. After one month of low-temperature dark culture, the growth rate of *M. aeruginosa* was only approximately 87.1% of its original rate [22]. However, there have been no studies on cyanobacterial response and recovery after prolonged darkness.

Maintaining a cold chain transport at 4 °C is both challenging and costly in remote areas requiring ecological modification. Moreover, organisms intended for bioenergy reserves should not require extra energy for temperature preservation. Therefore, studying cyanobacterial growth and storage capacity at ambient temperature is imperative. In this study, unlike prior research, our experiment focused on dark storage without the need for cold conditions, extending the darkness duration to one year (360 days) and focusing on the recovery ability of cyanobacteria after prolonged dark treatment periods. This study addresses a research gap regarding the recovery of cyanobacteria after long dark exposure without cold conservation. Two cyanobacteria species, *M. aeruginosa* and *Pseudanabaena foetida*, were treated in darkness for a year, and their recoverability was evaluated monthly. Significant changes in the stress and growth rates of both cyanobacteria species were investigated.

## 2. Materials and Methods

### 2.1. Cyanobacteria Strains and Cultivation

Strain *Microcystis aeruginosa* (Kützing) Lemmermann (NIES 111) and *Pseudanabaena foetida* Niiyama, Tuji and Ichise var. *intermedia* Tuji and Niiyama (NIES 512), purchased from the National Institute for Environmental Studies (Tsukuba, Japan), were cultured in autoclaved flasks containing BG-11 medium at 25 °C in an incubator (MIR-254, Sanyo, Tokyo, Japan). The light was provided using cool white LED lamps with 25–30 μmol photons m^−2^ s^−1^ photosynthetically active radiation (PAR) with 12 h light:12 h dark photoperiodicity. The concentration of *M. aeruginosa*, which can exist in unicellular or small colonies in the lab condition, can be approximated by measuring its optical density at 730 nm (OD_730_). *P. foetida* and large *M. aeruginosa* colonies cannot be measured with OD_730_. *M. aeruginosa* cultures with an OD_730_ value of ~0.1, using a spectrophotometer (UVmini-1240, Shimadzu, Tokyo, Japan), were used as experimental samples, and the original cell counts were approximately 2 × 10^5^ cells/mL. Small-colony cyanobacteria, *P. foetida*, was left to stand for 30 min, and the supernatant with suspended cells was taken as the original sample. The extracted supernatant was assessed through five consecutive OD_730_ measurements, and the concentrations were all around 0.08, which ensured the uniform distribution of suspended cells.

### 2.2. Experimental Design

Prepared cyanobacterial solutions of both *M. aeruginosa* and *P. foetida* were divided into 39 samples of 15 mL centrifuge tubes. Each tube contained 2 mL of the original samples and 8 mL of the BG-11 culture solution to ensure that the samples would not be affected by insufficient nutrients during the maximum one-year dark incubation.

At the beginning of this study, three tubes for each cyanobacteria species were taken out to measure the physicochemical parameters of the initial group. The remaining 72 tubes were encased in aluminum foil and stored in two dark boxes in a light-free incubator, maintained at 22–25 °C. These darkness-treated samples underwent 12 different treatment durations, spanning from 30 to 360 days. After each 30-day dark period, three tubes of both *M. aeruginosa* and *P. foetida* samples were transferred to autoclaved conical flasks and re-cultured for another 30 days. This cultivation used 150 mL of BG-11 solution under a light intensity of 25–30 μmol photons m^−2^ s^−1^ PAR with 12/12 light duration and the original temperature. The initial OD_730_ of *M. aeruginosa* culture and *P. foetida* supernatant was 0.012 ± 0.001, and the initial cell count of *M. aeruginosa* was approximately 1333 cells/mL. During the re-culture period, the samples were shaken manually twice daily. After the re-culturing, samples were collected. Eighteen cell pellets were prepared from each species. One mL of culture was added to a two mL centrifuge tube and centrifuged at 13,500× *g* for 15 min at 4 °C to prepare the cell pellet (Tomy MX-105, Digital Biology, Tokyo, Japan). The cell counts, optical density, and chlorophyll fluorescence were measured using the remaining samples in the flasks on the sampling day.

### 2.3. OD_730_ and Cell Counts Analysis

The measurement of OD_730_ and cell count only applies to *M. aeruginosa* as it can be unicellular or gather in small colonies, whereas *P. foetida* exists in long colonies entangled with each other.

OD measurements are commonly used for unicellular or small colonies of cyanobacteria to measure the cell growth rate rapidly. The cell growth rate can be monitored through changes in the OD value, as shown in Equation (1) [23].

(1) $$\mu \propto \frac{{\log_{10}OD}_{t}-{\log_{10}OD}_{0}}{t}$$

where *μ* is the cell growth rate;

*t* is days since inoculation;

${OD}_{t}$ is optical density after *t* days;

${OD}_{0}$ is optical density when *t* = 0.

In this study, *t* = 30 days. The optical density was measured at 730 nm and the average ${OD}_{0}$ was 0.012. Thus, Equation (1) can also be written as follows:

(2) $$\mu \propto \log_{10}\frac{{OD}_{730}}{0.012}$$

where μ is the cell growth rate;

${OD}_{730}$ is the optical density measured at 730 nm after 30 days of re-culturing.

The OD_730_ and cell counts were measured on each sampling day, and 10 μL of a well-shaken *M. aeruginosa* solution was added to a hemocytometer (C-Chip, NanoEnTek, Hwaseong-si, Gyeonggi-do, Republic of Korea) using an automated cell counter (LUNA, Logos Biosystems, Anyang-si, Gyeonggi-do, Republic of Korea) to determine the density of *M. aeruginosa* cells.

### 2.4. Chlorophyll Fluorescence Analysis

The re-cultured samples were subjected to chlorophyll fluorescence (ChF) parameters on the collection day. Samples were subjected to 30 min of dark adaptation and ChF measurements by adding 30 mL to 5 cm diameter Petri dishes. For the *P. foetida* culture, cyanobacterial colonies suspended in the upper part of the solution were collected. The chlorophyll fluorescence parameters of the samples were quantified using the ChF imaging technique (Handy FluorCam-FC 100-H, Photon Systems Technology, Brno, Czech Republic), and photosystem efficiency (Fv/Fm) and nonphotochemical quenching (NPQ) were measured.

### 2.5. Hydrogen Peroxide Concentration Analysis

The H_2_O_2_ measurement followed the titanium(IV)-based method with modifications [24]. Cyanobacterial cell pellets were mixed thoroughly with 1 mL of 0.05 mM phosphate buffer (pH 6.5) and centrifuged at 10,000× *g* for 10 min at 4 °C for H_2_O_2_ extraction. A mixture of 0.1% titanium (IV) sulfate in a 20% H_2_SO_4_ (*v*/*v*) solution was prepared for analysis. The supernatant was mixed with the titanium (IV) sulfate at room temperature (23–25 °C) and the optical absorbance was measured at 410 nm, with an extinction coefficient of 0.69 mM cm^−1^, to estimate the H_2_O_2_ concentration.

### 2.6. Chlorophyll-a, Carotenoid, and Total Protein Content Analysis

The analysis of chlorophyll-a (Chl-a) and carotenoid content followed the ethanol-thermal method with modifications [25]. The collected samples were added to 1 mL of 95% ethanol and heated in a water bath at 75 °C for 10 min after mixing well. After sufficient extraction, the samples were centrifuged again at 13,500× *g* for 10 min at 4 °C, and the supernatant was collected. The absorbance of the supernatant was measured at 665, 649, and 470 nm. Chlorophyll-a and carotenoid contents were calculated using Equations (3) and (4) [26,27], respectively.

(3) $$C_{a}=13.95A_{665}-6.88A_{649}$$

(4) $$C_{x\cdot c}=\frac{1000{A_{470}+811.74A}_{665}-2851.30A_{649}}{245}$$

where $C_{a}$ is chlorophyll-a content;

$C_{x\cdot c}$ is carotenoid content;

$A_{665}$, $A_{649}$, and $A_{470}$, are the optical densities of the extracts at wavelengths 665, 649, and 470 nm, respectively.

The total protein content was analyzed using the Bradford method with modifications [28]. Pigment-removed cells (samples used for Chl-a extraction) were mixed with 0.5 mL of 0.5 mM NaOH and heated in a water bath at 70 °C for 10 min. The treated cells were centrifuged at 10,000× *g* for 10 min at 4 °C. The supernatant was collected and mixed with 1 mL of Bradford reagent (Wako Chemical, Tokyo, Japan) for 10 min, and the optical density of the mixed solution at 595 nm was measured. Total protein content was calculated using a standard curve measured before the experiment.

### 2.7. Phenotype Observation

To observe the change in the phenotype of both *M. aeruginosa* and *P. foetida* colonies, ten microliters of each re-cultured cell suspension were observed under a 400× magnification digital microscope (ZEISS Axiolab 5, Carl Zeiss, Tokyo, Japan).

### 2.8. Supplementary Experimental

After 12 months of re-culturing had been completed, three sets of storage samples which had been subjected to 13 months of dark treatment were incubated. Simultaneously, three sets of freshly cultured healthy cyanobacteria were also incubated. This was performed to determine if the growth of *M. aeruginosa* had been affected by 13 months of dark treatment. Due to inconsistencies in their initial concentrations, the cell counts of the two samples were not measured on the same day. Changes in sample concentrations over 12 days, starting from 2 × 10^5^ cells/mL, were recorded and presented as the experimental results.

### 2.9. Data Analysis

For *M. aeruginosa*, the OD_730_, cell counts, and ChF data showed anomalous values caused by incubation and measurement errors in the seventh and eleventh months, with high standard errors between parallel groups that were not statistically significant. Furthermore, data analysis of *P. foetida* also showed abnormalities in the fourth month. A rapid power failure in the incubator and a sterile processing error of the flasks contributed to the sample abnormalities. Therefore, some non-normal data were discarded from the Section 3. The interpolation method was used to calculate the data for the missing months.

All experiments were performed in triplicate, and three samples were collected from each replicate. Six samples were used for cell count calculation. For other parameters, at least nine independent analyses were performed. For chlorophyll-a, carotenoid, total protein content, and H_2_O_2_ analysis, one-way analysis of variance (one-way ANOVA) and unpaired *t*-test were performed using IBM SPSS Statistics, Version 25 (IBM, Armonk, NY, USA). Microsoft Excel 2019 (Microsoft, Washington, USA) was used for regression relations and data visualization. Differences were considered significant at (*) *p* < 0.05 and (**) *p* < 0.01, and different letters indicate significant differences (*p* < 0.05).

## 3. Results

### 3.1. Cell Growth and Chlorophyll Fluorescence Change

**Figure 1:**
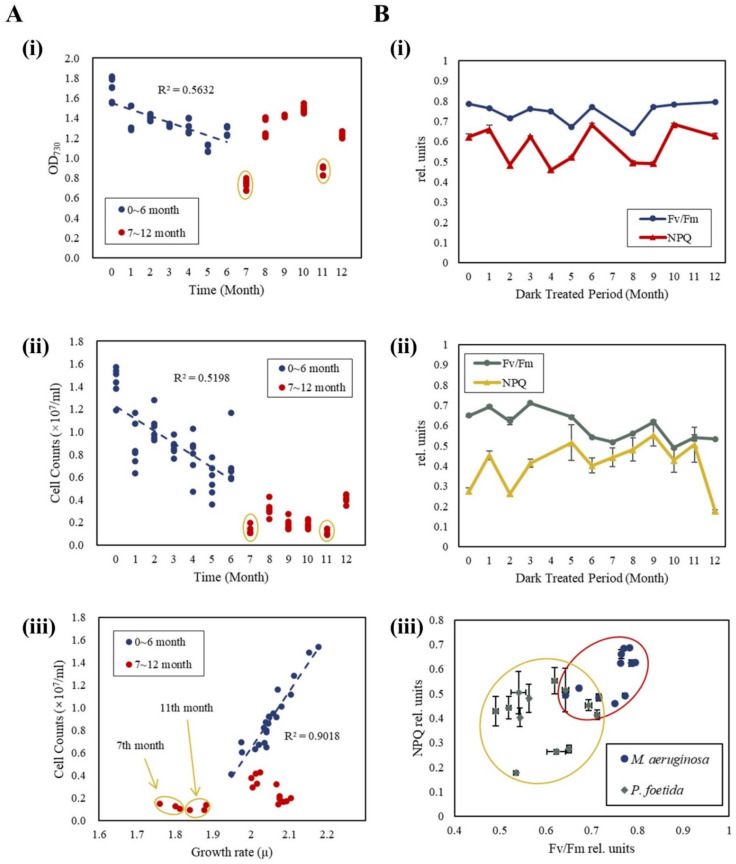
Cell counts and chlorophyll fluorescence (ChF) analysis in *Microcystis aeruginosa* and *P. foetida* re-cultured after the different dark treatment periods. Optical density (**A**(**i**)) and cell count, the blue dotted line represents the result of linear regression. (**A**(**ii**)) changes in dark-treated *M. aeruginosa* and the linear regression relationship between growth rate and cell counts, the points encircled by the yellow circle are outliers in the data, and the blue dotted line represents the result of linear regression on cell counts and growth rate. (**A**(**iii**)). The Fv/Fm and NPQ values over time for *M. aeruginosa* (**B**(**i**)) and *P. foetida* (**B**(**ii**)). There is a large gap between the two species in the ratio of Fv/Fm to NPQ values, the yellow and red circles indicate the value ranges of two cyanobacteria species. (**B**(**iii**)). Error bars represent standard errors.

Optical density and cell count changes were specific to *M. aeruginosa*, given its colonies form. As shown in Figure 1A(i), a consistent decline in optical density was noted from the beginning up to the sixth month, reaching a low point in the seventh month (OD_730_ = 0.74). After seven months of dark treatment, the OD_730_ value had ranged from 0.74 (seven-month dark-treated group) to 1.49 (ten-month dark-treated group) with a mean of OD_730_ = 1.18 (Figure 1A(i)). A similar pattern was observed in cell count analysis. The number of *M. aeruginosa* cells per mL declined from 1.43 × 10^7^ (non-dark-treated group) to 0.72 × 10^7^ (six-month dark-treated group). After seven months, the range of cell count values fluctuated from 0.11 × 10^7^ cells (eleven-month dark-treated group) to 0.41 × 10^7^ cells (twelve-month dark-treated group) with a mean of 0.22 × 10^7^ cells per mL (Figure 1A(ii)). The cell growth rate μ (represented as $\log_{10}\frac{{OD}_{730}}{0.012}$) and cell counts maintained a linear regression relationship for samples treated with darkness from the first to the sixth month (*R*^2^ = 0.902). However, no significant correlation was found between the seventh and the eleventh months (Figure 1A(iii)). In the experiment, the OD value decreased to 44% of the initial group by the end, whereas the cell count decreased to 8% of its initial group, consistent with the logarithmic relationship shown in Equation (2).

Similar to the data observed in the relationship between OD_730_ and cell counts of *M. aeruginosa* (Figure 1A(iii)), outlier values were noted for the Fv/Fm and NPQ values of *M. aeruginosa* at the seventh and eleventh months. Concurrently, *P. foetida* showed abnormal data in its fourth month. Upon excluding these outliers (the seventh and the eleventh months for *M. aeruginosa* and the fourth month for *P. foetida*), ChF analysis revealed that the mean value of the maximum Fv/Fm for *M. aeruginosa* was at 0.75 ± 0.04 (Figure 1B(i)), and the mean value of the maximum Fv/Fm for *P. foetida* was at 0.59 ± 0.11 (Figure 1B(ii)); both remained in a healthy level for cyanobacterial photosynthesis capability [29]. The mean NPQ value for *M. aeruginosa* was 0.57 ± 0.10 (Figure 1B(i)), while for *P. foetida,* it was 0.41 ± 0.22 (Figure 1B(ii)). The Fv/Fm and NPQ values for *M. aeruginosa* were higher than *P. foetida* and classified clearly into two clusters (Figure 1B(iii)).

### 3.2. H_2_O_2_ Concentration, Chl-a Content, and Carotenoid Content

**Figure 2:**
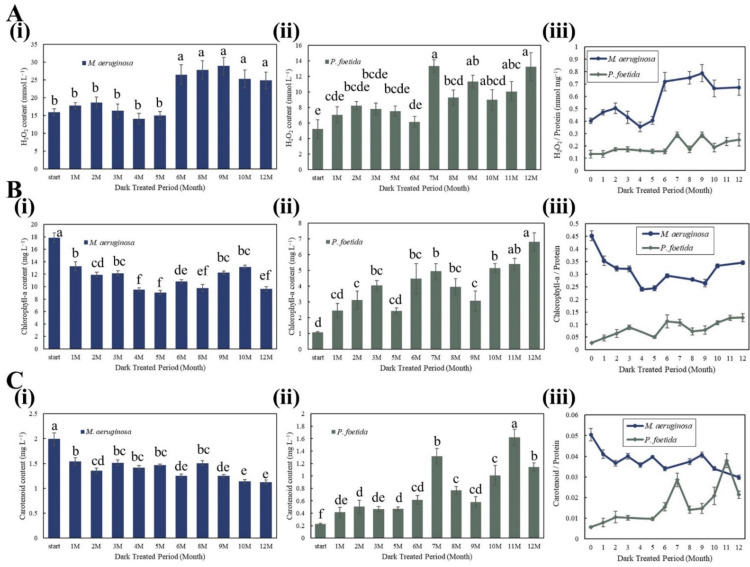
H_2_O_2_ concentration, chlorophyll-a, and carotenoid content in *M. aeruginosa* and *P. foetida* re-cultured following various dark treatment periods. H_2_O_2_ contents per one mL of *M. aeruginosa* (**A**(**i**)) and *P. foetida* (**A**(**ii**)) culture solution was measured and normalized by the total protein content to compare the H_2_O_2_ production for both species (**A**(**iii**)). Chl-a and carotenoid content in *M. aeruginosa* (**B**(**i**); **C**(**i**)) and *P. foetida* (**B**(**ii**); **C**(**ii**)), and the alteration of pigment per unit protein (**B**(iii); **C**(**iii**)), were also estimated in the same manner. Error bars represent standard errors. Different letters represent significant differences between dark treatment durations (one-way ANOVA, *p* < 0.05).

**Figure 3:**
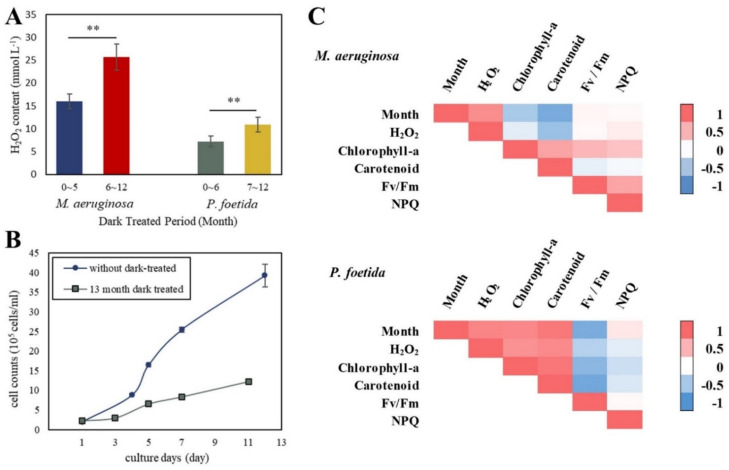
Supplementary experimental results and correlation analysis. The H_2_O_2_ levels in the two cyanobacteria were significantly different when grouped by their dark treatment period (**A**). The growth of re-cultured *M. aeruginosa* after a 13-month dark treatment period was slower compared to healthy cyanobacteria (**B**). Correlation analysis between specific parameters is presented in (**C**). Error bars represent standard errors. The differences were considered significant at (**) *p* < 0.01 (*t*-test). The correlation between parameters is expressed by the Pearson correlation coefficient *r*, in which *r* > 0 means positive correlation and *r* < 0 means negative correlation.

The H_2_O_2_ content changes in *M. aeruginosa* (Figure 2A(i)) and *P. foetida* (Figure 2A(ii)) showed a similar pattern; a more pronounced effect on H_2_O_2_ production was seen with an extended period of dark processing. The H_2_O_2_ content in *M. aeruginosa* cells had an extreme increase of 76.6% between the fifth month and the sixth month, while an increase of 117.0% in *P. foetida* cells between the sixth month and the seventh month was observed. Based on these differences, the following classification of both cyanobacteria via the length of the dark treatment period was suggested. *M. aeruginosa* can be divided into two groups: 0–5 months and 6–12 months, and *P. foetida* can be divided into two groups: 0–6 months and 7–12 months. Within the groups, no significant differences were found (one-way ANOVA, *p* > 0.1); however, significant differences between the groups were identified (*t*-test, *p* < 0.01) (Figure 3A). It was revealed that the H_2_O_2_ content per unit protein in *M. aeruginosa* cells was, on average, 148.5% higher than that in *P. foetida* (Figure 2A(iii)).

In *M. aeruginosa*, a slight decline over the dark treatment period was observed for both Chl-a and carotenoid content. It was shown that there was a sharp drop between the initial group (non-dark-treated group) and the one-month dark-treated group (Figure 2B(i),C(i)). In contrast to *M. aeruginosa*, a gradual increase in both Chl-a and carotenoid contents was seen in *P. foetida*. The lowest Chl-a and carotenoid contents of *P. foetida* appeared in the initial group, with the peak for Chl-a in the last month (Figure 2B(ii)) and the peak for carotenoid in the eleventh month (Figure 2C(ii)). It was indicated that the Chl-a content in *M. aeruginosa* cells was much higher than in *P. foetida* cells (Figure 2B(iii)), matching the results of the Fv/Fm value mentioned above. However, regarding the variation in carotenoid content per unit protein, higher initial values were seen in *M. aeruginosa* compared to *P. foetida*, but starting from the sixth month, an increase in carotenoid content in *P. foetida* cells was observed, with the highest point in the seventh and eleventh months.

### 3.3. Correlation Analysis

For the two cyanobacterial samples, the correlations between the six parameters are summarized in Figure 3C and displayed in red and blue. The darker color indicates a stronger correlation. Positive correlations between the two values are indicated in red and negative correlations in blue (Figure 3C). The H_2_O_2_ concentration in *M. aeruginosa* was positively correlated with the duration of the dark treatment period (*r* = 0.76). In contrast, Chl-a (*r* = −0.50) and carotenoids (*r* = −0.80) decreased with an increase in the duration of dark treatment. In *P. foetida*, Fv/Fm was significantly negatively correlated with the dark treatment period (*r* = −0.8), H_2_O_2_ concentration (*r* = −0.4), and carotenoid content (*r* = −0.70). At the same time, H_2_O_2_ concentration was positively correlated with the dark treatment period (*r* = 0.82) and carotenoid content (*r* = 0.78). In both cyanobacteria, chlorophylls and carotenoids showed an extremely high correlation (*r* = 0.61 for *M. aeruginosa* and *r* = 0.91 for *P. foetida*).

A more positive response to an increasing duration of dark treatment was exhibited by *P. foetida* than *M. aeruginosa*. The main differences in the responses of the two cyanobacteria to stress were highlighted by changes in Chl-a and carotenoid content.

### 3.4. Supplementary Experiment

From the second day onwards, the cell counts of the two groups of samples showed a clear difference. The cell counts of the samples without dark treatment increased steadily from the second day, with a rapid proliferation observed between the fourth and fifth day. In contrast, the dark treatment samples showed no increase in concentration from the first to the third day and gradually increased from the third day onwards. The results show that the growth rate of *M. aeruginosa* in dark culture was much lower than that of the healthy sample. The final cell concentration of *M. aeruginosa* without dark treatment was 195.5% higher than that of the thirteen-month dark-treated sample (Figure 3B).

### 3.5. Phenotype Observation

The prolongation of the dark processing period was not reflected in the microscopic photographs of the *M. aeruginosa* cells. No difference was observed between the initial group and the dark-treated groups through microscope photos. From the first month, both healthy and unhealthy cells were present in the *M. aeruginosa* samples. By the twelfth month, the prevalence of unhealthy cells appeared to be higher compared to the first and sixth months. However, the microscopic images served only for sampling observation, so the exact number of unhealthy cells remains unquantified.

**Figure 4:**
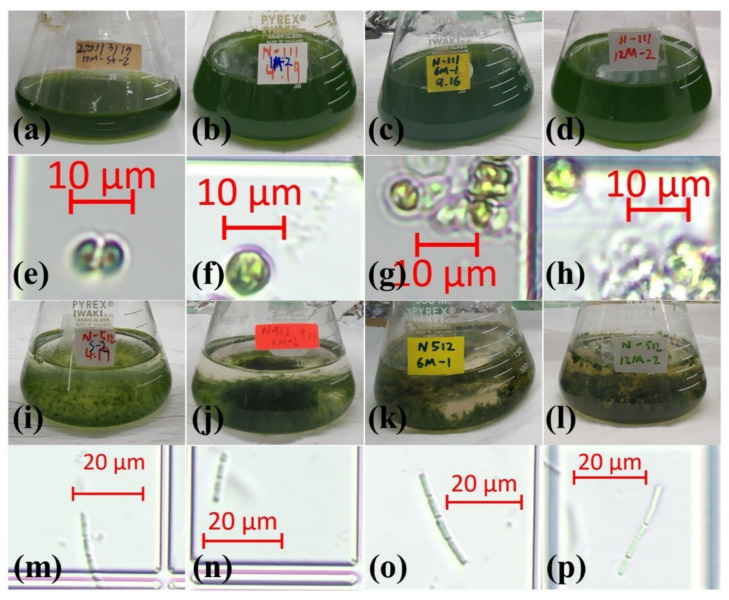
Phenotypic observation of experimental samples. (**a**–**d**). *M. aeruginosa* initial culture, one-month dark-treated culture, six-month dark-treated culture, and twelve-month dark-treated culture. (**e**–**h**). Microscope photos of *M. aeruginosa* cultures at initial culture, one-month dark-treated culture, six-month dark-treated culture, and twelve-month dark-treated culture. (**i**–**l**). *P. foetida* initial culture, one-month dark-treated culture, six-month dark-treated culture, and twelve-month dark-treated culture. (**m**–**p**). Microscope photos of *P. foetida* cultures at initial culture, one-month dark-treated culture, six-month dark-treated culture, and twelve-month dark-treated culture.

In contrast, compared to the initial group, dark treated *M. aeruginosa* cells more easily formed colonies and *P. foetida* cells were substantially larger. Throughout the twelve months of samples, both healthy and unhealthy cells were identified in two species, with no visible damage attributable to the dark treatment. Comparing the sample photos from the first month to the last month, it can be seen that the solution of *M. aeruginosa* has a slight tendency to become lighter in green color, while the solution of *P. foetida* was yellow, which matched the Chl-a and carotenoid results (Figure 4).

## 4. Discussions

### 4.1. Recovery from Prolonged Darkness Is Time-Dependent

Darkness is an influential factor affecting cyanobacterial growth and metabolism. A previous study has evidenced notable impacts on cyanobacteria responses after 30 days of darkness treatment [30]. In this study, the response of *M. aeruginosa* to dark treatment was nonlinear and exhibited phased variations. The H_2_O_2_ concentration increased abruptly when the dark treatment time exceeded the threshold. A similar response was also reported in *M. aeruginosa* when temperature conditions were changed and the environmental stress suddenly increased after 72 h of incubation temperature change [31]. This demonstrates that *M. aeruginosa* might adopt different coping mechanisms in response to long-term and short-term stress. However, the increase in H_2_O_2_ concentration over time in *P. foetida* was less than that in *M. aeruginosa*, indicating a lesser effect of darkness on *P. foetida*. A possible explanation is the increased carotenoid content, which acts as a scavenger of endogenous H_2_O_2_-induced oxygen species [32], especially for *P. foetida*, which shows a clear increase in carotenoid. For *M. aeruginosa*, the changes in H_2_O_2_ levels were more dramatic (Figure 3A). Microscope photos show (Figure 4g,h) that, starting at six months, *M. aeruginosa* cells are more likely to form colonies. Furthermore, cells within larger colonies appear more resistant to long-term, high-concentration H_2_O_2_ exposure, which may be related to the secretion of catalase (CAT) and glutathione (GSH) [33]. This finding indicates that both *M. aeruginosa* and *P. foetida* can recover after prolonged dark storage and can be reserved as bioenergy after complete global darkness conditions [20]. However, different storage conditions apply to different species, as the response of both cyanobacteria to darkness and temperature varies with time. Further research to determine the recovery under combined extreme conditions and with different species will provide more revealing results.

### 4.2. Recovery Is Affected by the Darkness Duration

In this study, we observed that cyanobacteria rapidly recovered from dark treatment. This is consistent with previous studies indicating that cyanobacterial photosynthesis diminishes in darkness but can revert during the recovery period [30]. However, contrasting earlier studies, our comparison of varying dark treatment durations revealed that extended periods hinder cyanobacterial recovery. The cell density of recovered *M. aeruginosa* samples treated for 12 months was lower compared to those treated for just one month. The decreased density during the re-culture period might be attributed to two reasons. Firstly, the dark treatment resulted in the death of some *M. aeruginosa* cells, leading to a reduced cell concentration before re-culture and a smaller base for re-cultivation. Second, the growth capacity of *M. aeruginosa* changed, leading to a slower growth of *M. aeruginosa*. We continuously monitored cell concentration changes and compared their growth over a 12-day period from the same initial concentration (2 × 10^5^ cells/mL). The non-dark-treated group exhibited a more rapid growth rate compared to the dark-treated group. This suggests that prolonged dark treatment can have prolonged effects on the growth of *M. aeruginosa* in the short term (within one month). Still, this does not exclude the possibility that some *M. aeruginosa* cells might die due to dark treatment. Future research will delve into the recovery conditions of cyanobacteria after prolonged darkness to assess the duration needed to achieve peak growth rates.

The combined effects of low temperature and dark storage on *M. aeruginosa* were studied by previous researchers [22]. It was noted that *M. aeruginosa* underwent a 12.9% ± 2.6% cell loss after one month of dark treatment compared to the control group. In other studies, researchers have pointed out that *M. aeruginosa* has vernalization effects and that low-temperature storage accelerates cyanobacterial recovery [21]. Some researchers have suggested that the dark environment increases the level of guanosine 3′—diphosphate 5′—diphosphate (ppGpp), further inhibiting the growth of cyanobacteria [18]. Combining these studies, we can infer that the recovery rate of cyanobacteria decreases after dark storage, which also coincides with the results of the present study.

### 4.3. M. aeruginosa Received a Greater Influence from Darkness Than P. foetida

As previously indicated, re-cultured *P. foetida* produced less H_2_O_2_ than *M. aeruginosa*. Correlation analysis revealed a positive association between H_2_O_2_ content and dark treatment duration for *M. aeruginosa*, whereas other parameters showed a negative correlation or no significant association (Figure 3C). A study on *M. aeruginosa* revealed that light is an essential factor affecting H_2_O_2_ decomposition in *M. aeruginosa* cells, and the difference in the promotion of catabolism in cells with and without light was evident at more than 24 h [34]. Other studies also have suggested that in vivo, H_2_O_2_ has a more pronounced effect on *M. aeruginosa* than on other cyanobacteria or plants [35], but *M. aeruginosa* cells exhibit enhanced immunity under in vitro H_2_O_2_ conditions [36]. Therefore, the decrease in cell growth, Chl-a, carotenoid, and other physiological indicators may result from the increased H_2_O_2_ content in recovering cells, which results from changes in cell physiology during adaptation to prolonged darkness exposure.

On the other hand, the Fv/Fm value of *P. foetida* showed a more pronounced decrease with darkness but recovered to healthy levels for cyanobacteria [29]. Like *M. aeruginosa*, there was a positive correlation between the H_2_O_2_ content and the duration of dark treatment. However, Chl-a and carotenoid contents in dark-treated *P. foetida* were higher than those without dark treatment. Moreover, micrographs also showed that dark-treated cells were larger than those without dark treatment. The increased carotenoid content in *P. foetida* enhanced its ability to scavenge H_2_O_2_ compared to *M. aeruginosa*. This suggests that even after complete darkness, *P. foetida* might sustain growth slowly or that darkness can induce a more rapid recovery of *P. foetida*. Further research is warranted to validate this theory. By evaluating H_2_O_2_ content, cell growth, and pigmentations, it appears that *M. aeruginosa* experienced greater suppression under dark conditions compared to *P. foetida*.

Differences in the responses of the two cyanobacterial species to darkness may be due to their different colonization methods. Several studies suggest that the light absorption and utilization capabilities of cyanobacteria are tied to their colony size and shape. Cyanobacteria can adjust their colony size based on external light fluctuations, mitigating the self-shadowing that intensifies with increasing size [37]. Variations in self-shadowing and light absorption might result in their recovery efficiencies. On the other hand, cyanobacteria in colonies, including both *M. aeruginosa* and *P. foetida*, with a heightened relative electron transport rate (rETR_max_) are more resistant to darkness than in single cells [38]. Therefore, *P. foetida,* which always form bigger colonies, may be more resistant to darkness than *M. aeruginosa*, even though *M. aeruginosa* forms tiny colonies [39]. Additionally, the colonization methods of cyanobacterial cell colonies is also a factor of their adaptation to environmental factors [40]. Therefore, circular colonized *M. aeruginosa*, and filamentous colonized *P. foetida* respond to darkness differently. Furthermore, factors like the stability of *psb*A, which encodes D1 protein, the core protein of photosystem (PS) II, transcripts affected by darkness [41], and the difference in genetic factors between the two species may also lead to differential responses.

## 5. Conclusions

In this study, *M. aeruginosa* and *P. foetida* were observed for their ability to recover after up to one year of dark treatment. In contrast to previous studies, we extended the dark treatment period to one year, comparing the growth of cyanobacteria across various dark treatment durations. Rather than focusing on the stress received by cyanobacteria in a dark environment, this study emphasized observing the re-culture of cyanobacteria once removed from the dark conditions. Previous to this research, the recovery capacity of cyanobacteria after extended darkness exposure has not been studied, making this a pioneering effort in the field. Regarding the duration of darkness, previous studies showed that different dark periods of 60 h [42], 8 days [18], and 30 days [26,27] had effects on both the growth and stress response of cyanobacteria. However, because of the different treatment duration and conditions, the outcomes of the studies cannot be compared with each other. Thus, the present study highlights the need for further research to distinguish species-specific responses to darkness. It also emphasizes the potential of using cyanobacteria as a bioreserve for recovery from catastrophes, space colonization, and other advanced human activities. Further research on the molecular and physiological mechanisms of dark adaptation and recovery should be conducted.

## Data Availability

No new data were created or analyzed in this study. Data sharing is not applicable to this article.
